# OnabotulinumtoxinA Is an Effective Treatment for Reducing the Interictal Burden in Patients with Chronic Migraine: A Prospective Observational Study

**DOI:** 10.3390/toxins17090463

**Published:** 2025-09-16

**Authors:** Alejandro Sánchez-Huertas, Oscar Camejo-Mas, Sebastian Garcia-Roldan, Rocio Alonso-Castillo, Lara Pulido-Fraiz, Andrea Higuera Ruiz de la Hermosa, Leonardo Portocarrero-Sánchez, Javier Díaz-de-Terán

**Affiliations:** 1 Neurology Department, Hospital Universitario La Paz, 28046 Madrid, Spain; ashuertas@salud.madrid.org (A.S.-H.); oscar.camejo98@gmail.com (O.C.-M.); sebastiangarcia1336@gmail.com (S.G.-R.); rocio.alonso.castillo@gmail.com (R.A.-C.); larapulidofraiz@gmail.com (L.P.-F.); anhiguerrh@gmail.com (A.H.R.d.l.H.); leonardo9493@gmail.com (L.P.-S.); 2 Hospital La Paz Institute for Health Research (IdiPAZ), Hospital Universitario La Paz, University Autónoma de Madrid, 28046 Madrid, Spain

**Keywords:** migraine, chronic migraine, OnabotulinumtoxinA, interictal burden, interictal symptoms, quality of life

## Abstract

Interictal burden (IB), defined as the symptoms and impairments that occur between migraine attacks, including cognitive dysfunction, photophobia, and fatigue, is recognized as a significant determinant of quality of life in patients. A prospective observational study was conducted. Patients diagnosed with chronic migraine (CM) and under treatment with OnabotulinumtoxinA (OnabotA) according to the PREEMPT protocol (every 12 weeks) were assessed at baseline and at 3, 6, 9, and 12 months. The primary endpoint was to evaluate the change in the IB measured with the Migraine Interictal Burden Scale (MIBS-4) and in the monthly migraine days (MMD). The secondary endpoint was acute medication use. This single-center study included 150 patients (91.3% female; median age 44 years). MIBS-4 scores were decreased by 29.1% at 3 months (8.47 to 5.97) and by 41.6% at 12 months (to 4.86; *p* < 0.001). IB-free status was achieved by 16 patients (10.7%). The most disabling baseline symptoms were photophobia (37%), fatigue (20%), and allodynia (18%), which reduced by 52%, 43%, and 39% at 12 months, respectively. MMD were reduced from 18.6 to 8.3 days at 12 months and triptan and analgesic intake decreased by 58.7% and 55.4%. OnabotA significantly reduced both IB and migraine frequency over 12 months, underscoring its relevance in CM management.

## 1. Introduction

In recent years, there has been a paradigm shift in the understanding and approaches to migraine. Traditionally, the focus has been on pain management during acute attacks, but the recent literature has begun to recognize the crucial importance of interictal burden and symptoms experienced between headache episodes, emerging as a crucial component in the comprehensive assessment of patients with migraine [1]. This new holistic approach recognizes that the impact of migraine extends beyond acute attacks, affecting patients’ lives even during pain-free periods.

Thus, interictal burden refers to the set of symptoms, functional limitations, and alterations in quality of life that persist between migraine attacks [2]. This burden can manifest in a variety of ways, including anticipatory chronic fatigue, anxiety, stigma, mood disturbances, motion sickness, or other sensory disturbances, such as persistent sensitivity to light or sound, and can even affect patients with few migraine days [3].

The assessment and management of the interictal burden have become essential components of a comprehensive approach to migraine treatment, recognizing that improvements in these aspects can have a significant impact on patients’ overall quality of life [4].

The importance of interictal burden lies in its ability to significantly affect patients’ quality of life, even on days when they do not experience headaches. Recent studies have shown that interictal burden is present in a significant proportion of patients with migraine, especially those with chronic migraine, implying that frequency and ictal burden are associated with interictal burden [3,5]. This burden has been observed to be as debilitating as migraine attacks, contributing substantially to the overall disability associated with the disease [6].

The assessment of interictal burden has been facilitated by the development of specific tools, such as the Migraine Interictal Burden Scale (MIBS-4), which allows for the quantification of the impact of migraine between attacks. This scale has proven to be valid and reliable for measuring interictal burden, providing a more complete view of a patient’s health status [7].

Despite the fact that there are multiple therapeutic options currently available for the treatment of various health conditions, the development and implementation of effective treatment strategies and individualized treatment for each patient are a fundamental priority in the management of chronic migraine, given that not all patients respond in the same way to medications that are already available on the market. In this sense, it is important to note that few treatments have been studied regarding their impact on the reduction in the interictal burden experienced by patients suffering from migraine, which is a crucial aspect to consider in their treatment. Addressing this research gap could lead to the identification of novel therapeutic approaches that not only alleviate acute migraine attacks but also enhance the overall quality of life by minimizing the frequency and severity of interictal symptoms. OnabotulinumtoxinA (OnabotA) is a serotype A botulinum neurotoxin with demonstrated efficacy in reducing both the frequency and severity of migraine attacks, as well as improving interictal symptoms and quality of life [8]. Compared with other botulinum toxins, such as abobotulinumtoxinA or incobotulinumtoxinA, OnabotA exhibits lower diffusion from the injection site, high potency per unit, and a well-characterized safety profile [9].

However, despite these advances, a significant gap remains in our knowledge of how different preventive treatments affect interictal burden over time. This gap is particularly notable in the context of chronic migraine, where the distinction between ictal and interictal periods may be less clear owing to the high frequency of attacks.

In this context, OnabotA is presented as a therapeutic option with the potential to address both the ictal and interictal aspects of migraine. The present study aimed to explore the impact of OnabotA on the IB of patients with CM, thus contributing to the evidence base for a more comprehensive and effective management of this debilitating condition. Few articles, with a limited number of patients, have addressed this IB issue [10].

The current study aimed to assess the effect of OnabotA in a cohort of patients with CM during an extended follow-up period. Specifically, it complements the scarce data on interictal treatment by evaluating its impact on the consumption of NSAIDs and triptans, frequency of migraine days, interictal symptoms, and quality of life as assessed by the MIBS-4 scale, aiming to provide further evidence on the efficacy of this treatment and its potential to alter various facets of the condition over time.

## 2. Results

**Figure 1:**
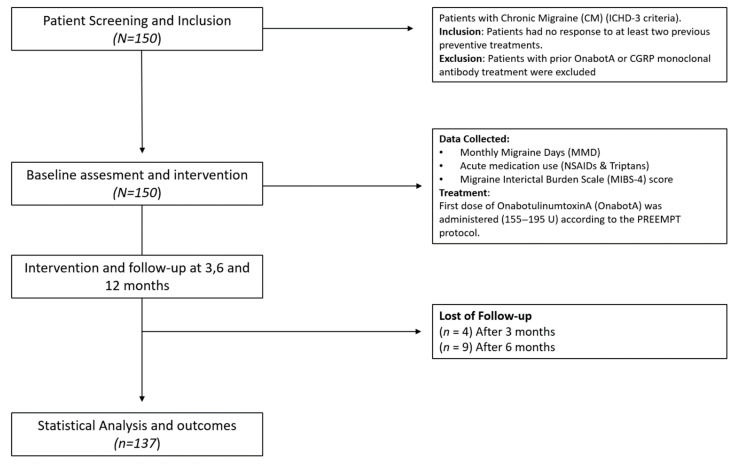
Flow diagram.

A total of 150 patients were included in the study, with a median age of 44 years (IQR: 37–52). Most of the participants were women (137 patients, 91.3%). The demographic and baseline patient characteristics are presented in Table 1 and Figure 1 shows the study flow diagram.

The study population had a history of multiple prior therapeutic attempts, with a median of three previous preventive treatments [IQR: 2–4].

A high prevalence of comorbidities was observed, emphasizing the clinical complexity of CM. Insomnia was the most common, affecting 36% of patients, followed by anxiety (26%), depression (22%), and fibromyalgia (11.3%). Additionally, 42% of patients reported undergoing psychotherapy.

Migraine with aura was observed in 58 (38.7%) patients. Among these, visual aura was the most prevalent (81%), followed by sensory aura (10%), a combination of visual and sensory aura (7%), and dysphasic aura (1%). These findings are consistent with the typical distribution of aura subtypes in the migraine population.

Pain was identified as the most bothersome symptom during migraine attacks by most patients (87.2%), followed by nausea (5.4%) and photophobia (4%).

Interictal symptoms also substantially contribute to the overall disease burden. Photophobia was the most debilitating interictal manifestation (37%), followed by fatigue (20%), allodynia (18%), dizziness (10%), osmophobia (5%), sonophobia (4%), and nausea (1%). These observations underscore the critical need to address interictal manifestations in therapeutic interventions for chronic migraine.

A total of 137 (91.3%) patients completed the 12-month follow-up, indicating a high adherence to OnabotA treatment throughout the study period.

### 2.1. Primary Endpoint: Interictal Burden Improvement

**Figure 2:**
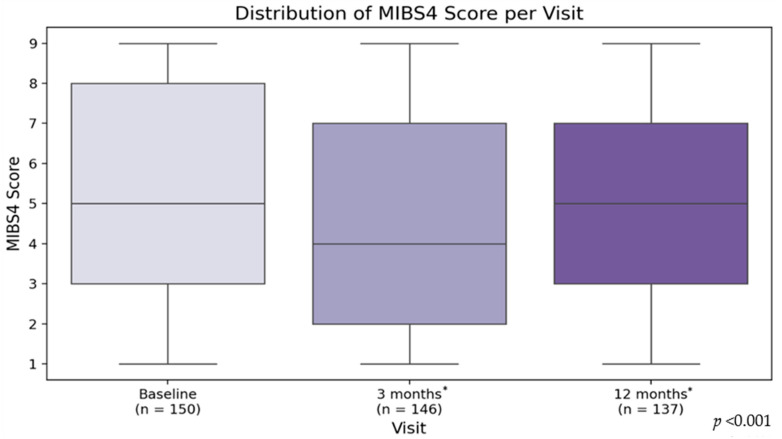
This figure shows the reduction in MIBS-4 scores from baseline at 3 and 12 months (* *p* < 0.001).

IB measured using the MIBS-4 scale showed a significant reduction in the treatment group. After three months of treatment with OnabotA, IB decreased by an average of 29.1% (from 8.47 to 5.97, *p* < 0.001). Over the following nine months, 55 patients (36.7%) maintained stable IB levels, while 89 patients (59.3%) continued to improve, reaching an overall 41.6% reduction by the end of the study (from 8.47 to 4.86, *p* < 0.001). The median IB score also showed a significant decline from 9 [interquartile range (IQR: 6–12] at baseline to 6 [IQR: 3–8] at 3 months and further to 5 [IQR: 2–7] at 12 months (*p* < 0.001), as seen in Figure 2. This reflects a substantial improvement in the symptoms and limitations of migraine. Furthermore, 14 patients achieved complete freedom from interictal burden over the course of the study, representing 9.3% of the initial study population (14/150).

Comparisons were made between patients with migraine with and without aura regarding the reduction in interictal burden, to determine if the presence of aura could be associated with a greater or lesser response to treatment. However, the results did not show statistically significant differences. 

### 2.2. Evolution of Headache Days and Treatment Response Rates

**Figure 3:**
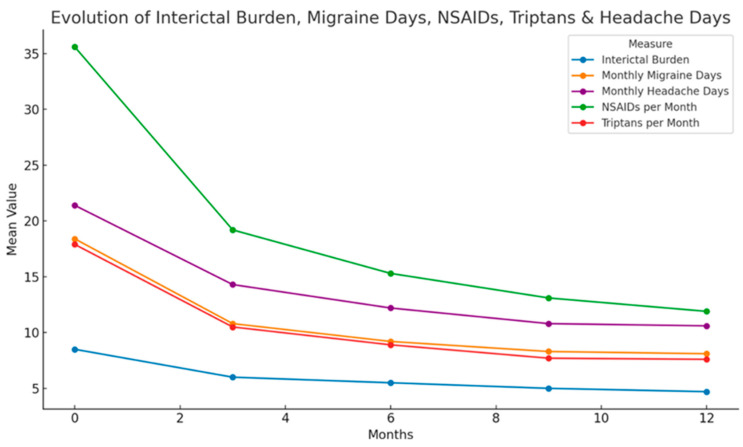
Evolution of primary and secondary outcomes during one year of OnbotA treatment, showing sustained benefit.

The mean number of migraine days (MMD) decreased significantly throughout the study period. At baseline, the mean MMD was 18.6, which decreased to 10.7 at 3 months and further decreased to 8.3 at 12 months. The median MMD showed a progressive decline from 16 days [IQR: 15–23] at baseline to 10 days [IQR: 6–15] at 3 months, 7 days [IQR: 4–12] at 6 months, and stabilized at 7 days [IQR: 4–11] at 9 and 12 months. This reduction was statistically significant (*p* < 0.001). The mean number of headache days (MHD) showed a marked reduction over the 12-month study period. At baseline, the mean MHD was 21.4 days, decreasing to 14.3 days at 3 months, 12.2 days at 6 months, 10.8 days at 9 months, and 10.6 days at 12 months. The median MHD followed a similar downward trend, declining from 19 days [IQR: 17–25] at baseline to 13 days [IQR: 9–18] at 3 months, 10 days [IQR: 6–15] at 6 months, and stabilizing at 9 days [IQR: 6–13] at both 9 and 12 months. This reduction was statistically and clinically significant (*p* < 0.001), indicating sustained improvement in headache frequency throughout the study period. The treatment outcomes during 1 year of treatment are summarized in Table 2 and Figure 3.

The overall treatment effectiveness was evaluated based on the percentage reduction in MMD and MHD, categorized into response rates of ≥30%, ≥50%, and ≥75%. At 3 months, 106 patients (70.6%) achieved a ≥30% reduction in MMD, with 86 patients (57.3%) maintaining this response at 12 months. A ≥50% reduction was observed in 59 patients (39.3%) at 3 months and 70 patients (46.7%) at 12 months. Finally, a ≥75% reduction was achieved in 22 patients (14.7%) at 3 months and 37 patients (24.7%) at 12 months. Regarding MHD, 113 patients (75.3%) achieved a ≥30% reduction at three months, with 91 patients (60.7%) maintaining this response at twelve months. A ≥50% reduction was observed in 66 patients (44.0%) at 3 months and in 78 patients (52.0%) at 12 months. Lastly, a ≥75% reduction in MHD was achieved in 27 (18.0%) at 3 months and 42 (28.0%) at 12 months. These findings suggest that the effectiveness of treatment in reducing headache frequency was sustained or even improved over time in a substantial proportion of patients.

Regarding pain intensity, among the 150 patients analyzed, approximately 116 (77.3%) experienced an improvement in headache intensity during the study period.

### 2.3. Analgesic and Triptan Consumption

A significant reduction in the use of analgesics and triptans was observed during the study period. NSAID consumption decreased from a median of 30 doses per month [IQR: 20–42] at baseline to 14 doses [IQR: 7–25] at 3 months, 10 doses [IQR: 5–20] at 6 months, and 7 doses [IQR: 4–13] at 12 months (*p* < 0.001). Similarly, triptan consumption declined from 15 doses [IQR: 7–25] at baseline to 8 doses [IQR: 3–14] at 3 months, 7 doses [IQR: 3–10] at 6 months, 6 doses [IQR: 3–10] at 9 months, and 5 doses [IQR: 2–10] at 12 months (*p* < 0.001). Overall, there was a 55.4% reduction in analgesic use (from 35.6 to 11.9, *p* < 0.001) and a 58.7% reduction in triptan use (from 18 to 7.42, *p* < 0.001).

## 3. Discussion

It is important to highlight that our findings not only reinforce the efficacy of OnabotA in alleviating the interictal burden associated with CM but also suggest a broader therapeutic impact on comorbid neurological symptoms. The significant reduction in interictal disability observed in this cohort supports the growing recognition that effective migraine treatment must address more than just the frequency of attacks. This multidimensional improvement contributes meaningfully to patient quality of life, which is often overlooked in clinical trials that focus solely on the reduction in monthly migraine days.

The results obtained in this study not only align with but also surpass those of previous research on the impact of OnabotA on interictal symptoms in patients with CM. The larger sample size of 150 patients and an extended follow-up period of 12 months revealed a substantial 41.6% reduction in interictal burden. This outcome is notably more significant than the findings reported by Argyriou et al. [9], who observed a 32% reduction in MIBS-4 scores in a smaller cohort of 70 patients over three cycles of treatment. Similarly, Alonge et al. [10] reported significant decreases in interictal burden among 52 patients treated for 6 months. Therefore, this study demonstrated more comprehensive and sustained improvement.

The extended duration and larger scale of this study allowed us to observe additional benefits that were not captured in short-term investigations. Nearly 10% of our patients became entirely free of interictal burden, a finding that underscores the potential for complete symptom resolution in some patients. Furthermore, it was noted that improvements were maintained and even enhanced beyond the initial treatment cycles, suggesting a cumulative benefit of OnabotA therapy over time. The administration of OnabotA requires precision, as dosing errors are difficult to reverse and may lead to unintended weakness or reduced efficacy. Strict adherence to standardized protocols, such as PREEMPT, is essential for safety. Emerging research on botulinum neurotoxin A inhibitors may provide future tools to better control the toxin’s effects and expand therapeutic options [11].

These results emphasize the long-term therapeutic potential of OnabotA in addressing not only the acute pain associated with migraines but also the broader spectrum of migraine-related disabilities that persist between episodes. Long-term studies have shown that OnabotA maintains its efficacy in reducing migraine frequency and interictal symptoms over extended periods, with no significant safety concerns. A five-year retrospective study confirmed sustained reductions in migraine frequency and IB with a favorable safety profile [12].

Thus, these findings provide a more comprehensive understanding of OnabotA’s efficacy in managing the full range of migraine-related symptoms beyond pain and highlight its potential as a long-term treatment strategy for CM patients.

Moreover, when comparing the results with those from studies on CGRP monoclonal antibodies, OnabotA showed superior or comparable reductions in the interictal burden. For instance, the CONQUER study evaluating galcanezumab in patients with prior treatment failure reported a mean MIBS-4 reduction of 1.9 points at 3 months [13], whereas our cohort showed a reduction of 2.50 points (29.5%) at the same time point and a further reduction of 3.61 points (42.6%) at 12 months. Likewise, the EMBRACE study, a real-world multicenter investigation of eptinezumab, reported a mean reduction of 2.1 points in MIBS-4 after 6 months of treatment [14], which was still slightly lower than the reductions observed in our study at both 3 and 12 months. However, we suggest that combining OnabotA and these therapies could address different pathways involved in migraine pathophysiology, potentially leading to improved efficacy in managing chronic migraine symptoms. OnabotA and CGRP mAbs target different migraine pathways, with OnabotA inhibiting C-fibers and CGRP mAbs preventing Aδ fiber activation. This complementary action may explain the enhanced efficacy observed with combination therapy [15,16]. This combination can significantly enhance the quality of life of patients and reduce the frequency of migraine attacks, and is particularly beneficial for patients with treatment-resistant CM, as it addresses multiple aspects of migraine pathophysiology [16,17]. However, despite its potential, there is currently a lack of evidence supporting widespread clinical guideline inclusion, and the high cost of such combinations poses feasibility challenges for patients [18].

These results are further supported by this study’s ability to complement and reinforce previous observations regarding the efficacy of OnabotA [9]. We offer robust real-world evidence from a broader clinical population, including individuals with higher rates of psychiatric comorbidities and previous treatment failure. These factors often limit the generalizability of smaller or more selective clinical trials; however, in this investigation, they enhanced the external validity of our findings. In the current therapeutic landscape, characterized by rapid innovation and personalized care, OnabotA continues to play a significant role. Its unique mechanism, which targets SNAP-25-mediated neurotransmitter release, differs fundamentally from that of CGRP antagonists, and may be especially advantageous in patients with comorbid muscle tension, allodynia, or insomnia, in which peripheral and central sensitization mechanisms are prominent [19].

In conclusion, despite the availability of newer agents, OnabotA remains the cornerstone of CM management. Its enduring efficacy, safety, and broader symptom relief, as confirmed by our results and supported by the growing body of literature [20], underscore its relevance in a multimodal, patient-centered approach to chronic migraine management. The long-term benefits we observed in interictal burden, coupled with sustained improvements in the MIBS-4 and other scales, highlight the integral role of OnabotA in the treatment of chronic migraine.

This study has several notable strengths that enhance its scientific value and contribute to CM research. A key strength lies in its comprehensive approach, which extends beyond the traditional focus on headache frequency to examine the interictal burden experienced by patients with chronic migraine. This holistic perspective provides a more nuanced understanding of how OnabotA treatment impacts patients’ overall quality of life. The incorporation of the Migraine Interictal Burden Scale (MIBS-4) in its Spanish version further bolsters the study’s robustness, offering a standardized and reliable measure of interictal burden that enhances the validity of the findings and facilitates comparisons with other studies. It is important to note that there is no officially validated version of this scale in our language; however, it has been used in some international multicenter clinical trials in recent years and is well received by patients because of its simplicity.

The study’s well-defined inclusion and exclusion criteria, based on the International Classification of Headache Disorders (ICHD-3) guidelines, ensured a clearly delineated patient population, thereby increasing its validity. The long-term follow-up period of one year, with multiple assessment points, allowed for a comprehensive evaluation of treatment effects over time, capturing both immediate and sustained changes in patients’ conditions. The sample size was larger than that of previous studies approaching this issue, and only three patients dropped out of the study. Additionally, the study’s use of multiple outcome measures, including headache frequency, medication use, and interictal burden, provided a multifaceted view of treatment efficacy.

However, our study had several limitations that warrant consideration. The absence of a control group limits the ability to definitively attribute the observed changes to OnabotA treatment rather than other factors or natural disease progression. Potential selection bias, particularly due to the exclusion criteria regarding previous treatments, may limit the generalizability of the results to a broader chronic migraine population. Additionally, potential confounding factors, such as lifestyle changes or concurrent treatments, should be addressed and accounted for in the analysis. As this was a single-center study, it may limit the generalizability of the results to other populations or healthcare settings. Nonetheless, the headache clinic functions as a tertiary public facility that accommodates individuals from various health sectors and diverse geographical locations within Spain, thereby mitigating this limitation.

## 4. Conclusions

Our findings demonstrate that OnabotA not only effectively reduces monthly migraine days, pain intensity, and the need for acute symptomatic treatment, but also significantly alleviates the interictal burden associated with chronic migraine. This multidimensional therapeutic impact translates into meaningful improvements in quality of life, often underrepresented in the traditional endpoints of migraine research. Notably, the sustained reduction in IB observed in this cohort, including symptoms such as photophobia, dizziness, fatigue, and allodynia, highlights the potential of OnabotA to address both the ictal and interictal phases of the disorder.

In contrast to many randomized controlled trials, this real-world study included a broader and more representative patient population, including individuals with high psychiatric comorbidities and prior therapeutic failures. The robust and durable improvements observed over 12 months reinforce the external validity and clinical applicability of our findings. Furthermore, the reduction in MIBS-4 scores in our cohort exceeded those reported in recent trials of CGRP monoclonal antibodies, including the CONQUER and EMBRACE studies, underscoring the potential comparative advantage of OnabotA in managing interictal symptoms.

Despite the rise in newer targeted therapies, OnabotA remains a cornerstone in the multimodal management of chronic migraine. Its unique mechanism of action, which modulates both peripheral and central sensitization pathways, offers a complementary profile to CGRP-targeted agents, particularly in patients with prominent interictal symptoms or complex migraine phenotypes.

Nevertheless, future studies are warranted to better elucidate the mechanistic underpinnings of interictal symptom improvement with OnabotA and identify biomarkers that may predict treatment response. Additionally, comparative head-to-head studies evaluating the impact of different therapeutic strategies on both ictal and interictal dimensions are needed to optimize individualized care.

In conclusion, these results strongly support the continued and strategic use of OnabotA in CM treatment, especially within comprehensive care models that recognize the importance of reducing the total migraine burden—not only the frequency of attacks but also the often-overlooked interictal suffering that significantly impairs daily functioning and well-being.

## 5. Materials and Methods

According to the International Recommendations for Observational Studies, items on the Strengthening the Reporting of Observational Studies in Epidemiology checklist were followed in this study [21]. This study was approved by the Ethics Committee of Hospital Universitario La Paz, Madrid (code PI-5734). We conducted a prospective cohort observational study with successive recruitment at a specialized headache clinic in La Paz University Hospital in Madrid, Spain. Patients with migraine were previously diagnosed by a headache specialist according to the ICHD-3 criteria [22]. The study period was from July 2023 to July 2024. Data were collected at five visits, including the baseline visit, when the first OnabotA dose was injected, and every three months over a one-year follow-up. Patients received OnabotA (155–195 U) every 12 weeks following the standardized PREEMPT injection paradigm [23], which involves 31–39 injection sites across the head and neck muscles, including the frontalis, corrugator, procerus, temporalis, occipitalis, cervical paraspinal, and trapezius muscles. Additional “follow-the-pain” injections were administered at the discretion of the treating neurologist. Each site received 5 U, with injections spaced approximately 1 cm apart.

**Figure 4:**
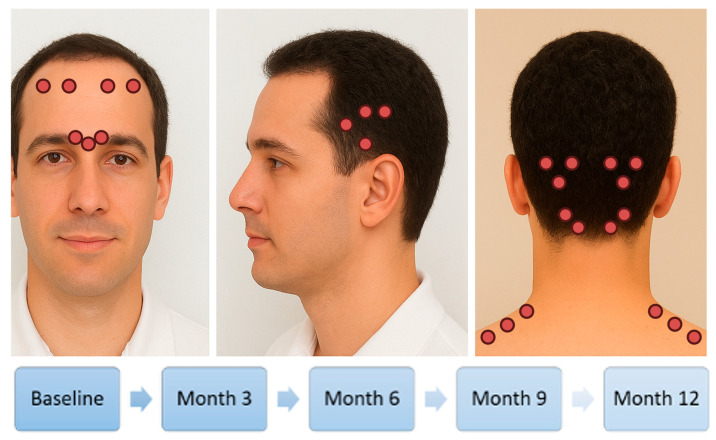
This figure illustrates the distribution of OnabotA injections and the schedule of administration.

To facilitate comprehension, we include a figure (Figure 4) showing the injection sites, localization, and timing strategy in a flow chart format.

### 5.1. Study Population and Eligibility

Adult patients (≥18 years) diagnosed with chronic migraine according to the ICHD-3 [22] criteria were included. The inclusion criteria were as follows:

- Diagnosis of chronic migraine according to the ICHD-3.
- Absence of response to at least two previous preventive treatments.
- The exclusion criteria were as follows.
- Current or previous treatment with OnabotA.
- Current or previous treatment with monoclonal antibodies against the CGRP.
- Diagnosis of episodic migraine.
- Pregnancy, breastfeeding, or a desire to become pregnant.
- Any formal contraindication per technical data sheet for the use of OnabotA.

### 5.2. Study Procedure

Patients attending the headache clinic were recruited if they met the abovementioned requirements. After obtaining informed consent, five face-to-face visits were scheduled over one year:

(1) Baseline visit;
(2) Visit at 3 months;
(3) Visit at 6 months;
(4) Visit at 9 months;
(5) Final visit at 12 months.

At each visit, 195 international units of OnabotA were administered following the PREEMPT paradigm.

### 5.3. Data Collection

At each visit, a trained researcher administered an ad hoc questionnaire that included the questions on the following:

1 Sociodemographic characteristics.
2 Clinical features of migraine:
  - Headache days per month or migraine;
  - Migraine days per month;
  - NSAIDs consumption per month;
  - Triptans consumption per month.
3 Psychiatric emotional comorbidity: Anxiety, depression, and insomnia were assessed using the Hospital Anxiety and Depression Scale (HADS).
4 Interictal symptoms described by the patient on a checklist.
5 The interictal burden was measured using the Migraine Interictal Burden Scale 4 (MIBS-4). To date, despite recent validations in languages closely related to Spanish (e.g., Portuguese) [24], this scale lacks a formally validated Spanish-language version. However, the Spanish translation has been used and accepted in international multicenter clinical trials [13] and in communications at various national and European scientific conferences [25]. The appropriate use of scales/PROMs in a patient’s native language is crucial to ensure responses accurately reflect the patient’s reality and to aid their comprehension of their condition [26]. Since the primary objective of this study was to assess the reduction in MIBS-4 scores, and given the absence of a Spanish validation, the scales were administered in person by neurologists with high English proficiency. This approach ensured the translation remained as faithful as possible to the scale’s original language. Any uncertainties patients had regarding question interpretation were clarified by the examiner.

As per usual clinical practice, patients maintained a paper or electronic diary during the OnabotA treatment period to register headache days and acute medication intake.

### 5.4. Sample Size

The study utilized all available patient data at the time of analysis. As preliminary data and prior studies investigating this specific mechanism of action were lacking, an initial sample size calculation was not performed. Furthermore, during 2024, a related study [9] demonstrated a significant decrease in MIBS-4 scoring with N = 70, supporting the adequacy of our final sample size.

### 5.5. Statistical Analysis

Data analysis was performed using the Statistics Package for Social Science (SPSS 23.00; IBM Inc., Armonk, NY, USA) and Julius AI (Caesar Labs, Inc., San Francisco, CA, USA, accessed on 18 December 2024 through https://julius.ai). Descriptive analyses were performed for all variables. A linear mixed model for repeated measures was used to assess changes in the interictal burden and other parameters over time. Statistical significance was set at *p* < 0.05. Nominal variables were reported as percentages and compared using a two-tailed Chi-square or Fisher’s exact test, when applicable. A 2-tailed Shapiro–Wilk test was applied to examine whether the ratio variables followed a Gaussian distribution. Ratio variables were reported as mean ± SD if they followed a Gaussian distribution; otherwise, they were represented as median ± interquartile range [p25–p75]. Statistical significance was set at *p*, 0.05 and the confidence interval was set at 95%.

## Figures and Tables

**Table 1:** Demographic and baseline patient characteristics collected in the study.

Category	Variable	Value
**Baseline Characteristics**	Age, Median (IQR)	44 (37–52)
	Female, n (%)	137 (91.3)
	Anxiety, n (%)	39 (26)
	Depression, n (%)	33 (22)
	Insomnia, n (%)	54 (36)
	Fibromyalgia, n (%)	17 (11.3)
	Interictal Symptoms, n (%)	116 (77.3)
	Previous Preventive Treatments, Median (IQR)	3 (2–4)
	Chronic migraine, n (%)	150 (100)
	Migraine with aura, n (%)	58 (38.7)
**Most Disabling Ictal Symptoms**	Pain, n (%)	130 (87.2)
	Nausea, n (%)	8 (5.4)
	Photophobia, n (%)	6 (4)
	Visual aura, n (%)	1 (0.7)
**Most Disabling Interictal Symptoms**	Photophobia, n (%)	38 (37)
	Fatigue, n (%)	24 (20)
	Allodynia, n (%)	19 (18)
	Dizziness, n (%)	11 (10)
	Osmophobia, n (%)	5 (5)
	Sonophobia, n (%)	4 (4)
	Nausea, n (%)	1 (1)

**Table 2:** Treatment outcomes during the study period.

	Baseline	3rd Month	6th Month	9th Month	12th Month
MMD, median [IQR]	16 (15–23)	10 (6–15)	7 (4–12)	7 (4–11)	7 (3–11)
MA, median [IQR]	30 (20–42)	14 (7–25)	10 (5–20)	8 (5–16)	7 (4–13)
MT, median [IQR]	15 (7–25)	8 (3–14)	7 (3–10)	6 (3–10)	5 (2–10)
IB, median [IQR]	9 (6–12)	6 (3–8)			5 (2–7)

## Data Availability

The original contributions presented in this study are included in the article. Further inquiries can be directed to the corresponding author.

## Abbreviations

The following abbreviations are used in this manuscript:

IB: interictal burden; OnabotA: Onabotulinum toxin A; ICHD-3: International Classification of Headache Disorders, 3rd edition; NSAIDs: non-steroidal anti-inflammatory drugs; MMD: monthly migraine days; CM: chronic migraine; MA: monthly analgesic intake; MT: monthly triptan intake; HADS, Hospital Anxiety and Depression Scale; MIBS-4: Migraine Interictal Burden Scale 4.
